# Mass spectrometry-intensive top-down proteomics: an update on technology advancements and biomedical applications

**DOI:** 10.1039/d4ay00651h

**Published:** 2024-06-27

**Authors:** Tian Xu, Qianjie Wang, Qianyi Wang, Liangliang Sun

**Affiliations:** a Department of Chemistry, Michigan State University 578 S Shaw Lane East Lansing MI 48824 USA lsun@chemistry.msu.edu +1-517-353-0498

## Abstract

Proteoforms are all forms of protein molecules from the same gene because of variations at the DNA, RNA, and protein levels, *e.g.*, alternative splicing and post-translational modifications (PTMs). Delineation of proteins in a proteoform-specific manner is crucial for understanding their biological functions. Mass spectrometry (MS)-intensive top-down proteomics (TDP) is promising for comprehensively characterizing intact proteoforms in complex biological systems. It has achieved substantial progress in technological development, including sample preparation, proteoform separations, MS instrumentation, and bioinformatics tools. In a single TDP study, thousands of proteoforms can be identified and quantified from a cell lysate. It has also been applied to various biomedical research to better our understanding of protein function in regulating cellular processes and to discover novel proteoform biomarkers of diseases for early diagnosis and therapeutic development. This review covers the most recent technological development and biomedical applications of MS-intensive TDP.

## Abstract 2

Substantial progress in the technological development of mass spectrometry-based top-down proteomics enabled broad biomedical applications for bettering our understanding of proteoform function in modulating diseases and development.

## Graphical abstract

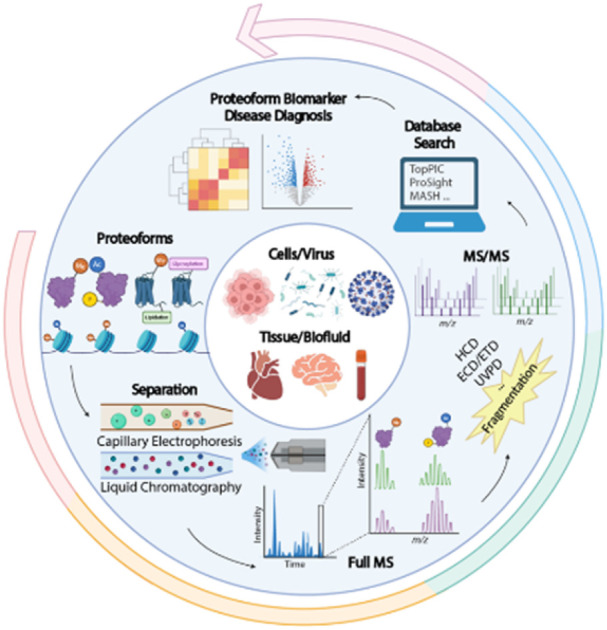

## 1. Introduction

**Fig. 1:**
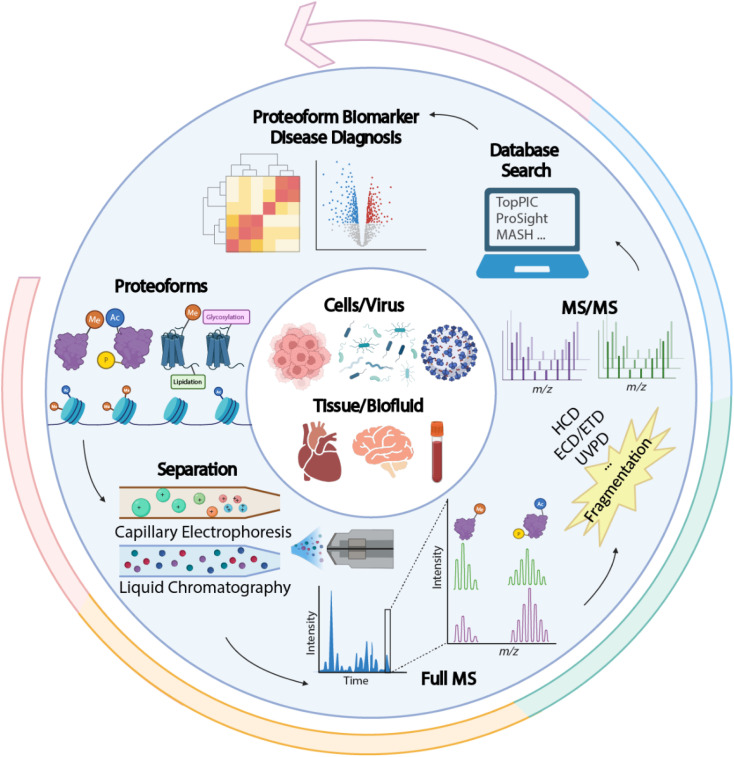
Schematic of a typical top-down proteomics (TDP) workflow, including proteoform extraction from various biological samples, separation by liquid chromatography (LC) or capillary electrophoresis (CE), mass spectrometry (MS) and tandem MS (MS/MS) *via* various gas-phase fragmentation techniques, and database search using bioinformatics tools for proteoform identification and quantification to discover new proteoform biomarkers of diseases. The figure is created using the BioRender and is used here with permission.

Proteins are direct functional participants in cells that link genotype and phenotype. Diverse forms of proteins (also termed “proteoforms”) can be derived from a single protein-coding gene due to gene mutations, RNA alternative splicing, and protein post-translational modifications (PTMs), resulting in various functional outcomes.^1–7^ Proteomics studies that interrogate sequence and PTM features of proteoforms are crucial for understanding their roles in complex biological and disease events. Compared to conventional bottom-up proteomics (BUP), which acquires regional information about proteoforms by mapping peptides, TDP deciphers the combinatorial PTMs and amino acid variations in individual proteoforms, enabling a comprehensive insight into the proteoform landscape of biological subjects. A universal MS-intensive TDP workflow includes protein extraction from biological samples, separation of proteoforms by liquid chromatography (LC) or capillary electrophoresis (CE), proteoform mass measurement and fragmentation by state-of-the-art mass spectrometers, and interpretation of proteomics data by advanced bioinformatics tools, Fig. 1.^8^

The complexity of proteoform identification arises from the need to identify and localize PTMs, determine amino acid sequences, and account for unknown or ambiguous genes.^9^ Based on the five-level classification system for proteoform identifications, around 40–60% of level 1 proteoforms can be identified without ambiguity.^10–12^ However, MS-intensive TDP is still considered challenging in multiple aspects. First, current large-scale TDP studies mainly focused on proteoforms smaller than 30 kDa. Complete characterization of large proteoforms is difficult due to coelution in separation and low sensitivity in MS detection. Second, MS-intensive TDP of membrane proteins is challenging due to their low solubility in MS-compatible buffers. Third, delineating heavily modified proteoforms (*e.g.*, histones) requires much more effort to improve separations, fragmentations, and bioinformatics tools for accurate PTM determination and localization. Fourth, achieving the identification (ID) of proteoforms of thousands of protein-coding genes from human cells in a single study is challenging for MS-intensive TDP due to the extremely high sample complexity. It has been estimated that more than one million proteoforms are present in the human body.^13^ Early large-scale MS-based TDP studies have generally achieved hundreds to thousands of proteoform IDs from several hundred genes in human cells. Lastly, more work is needed to improve the bioinformatics tools for proteoform ID and quantification to advance TDP in the abovementioned areas.^14–17^

During the last several years, MS-intensive TDP has made substantial progress in addressing these challenges. This process has been driven by drastic technological improvements in sample preparation, separation, MS instrumentation, fragmentation strategies, and bioinformatics tools. Several reviews have summarized the related topics in earlier years.^8,18–20^ In this review, we updated the most recent technological advancements in MS-intensive TDP, with a particular focus on large proteoforms, membrane proteins, heavily modified proteoforms, global proteoform profiling, high-throughput quantification, and bioinformatics tools. In addition, the review introduced the latest biomedical applications of MS-intensive TDP.

## 2. Technological development

### 2.1 Characterization of large proteoforms

Large proteoform ID from complex cell lysates has been dramatically restricted in global MS-intensive TDP studies. To date, most large-scale TDP efforts focused on proteoforms smaller than 30 kDa due to multiple technical challenges. For complex cell lysates, proteoform coelution in separation causes ionization suppression of large and low-abundance proteoforms.^21,22^ Also, the signal-to-noise (S/N) of proteoforms from MS detection decreases exponentially with increasing proteoform mass, due to a much broader charge state and isotopic distributions.^23,24^ In terms of MS instrumentation, inadequate transmission and mass resolution impede large proteoform IDs. Finally, limited backbone cleavage coverage of large proteoforms using conventional collision-based fragmentation methods constantly causes problems in precise sequence ID and PTM localization.^25,26^

Sample fractionation has been extensively applied in recent years to reduce sample complexity, which significantly benefits the downstream characterization of large proteoforms with top-down MS. Size exclusion chromatography (SEC) has been considered as a practical approach to fractionate proteoforms by size, enabling better characterization of large proteoforms. Coupling serial size exclusion chromatography (sSEC) fractionation and reversed-phase liquid chromatography (RPLC)-MS allowed a 15-fold increase in the number of detected proteoforms above 60 kDa.^25^ Further application of sSEC-RPLC-MS and MS/MS with Proteoform Suite software^17^ achieved the ID of large proteoforms up to 140 kDa from human heart samples.^26^ The 140 kDa protein (endogenous human cardiac myosin binding protein C) was identified by top-down MS for the first time. Most recently, the ID of intact large proteoforms (myosin heavy chain, ∼223 kDa) from single cells (single muscle fibers, SMFs) was reported *via* advanced RPLC-MS-based TDP.^27^ The Zhang group developed an SEC-RPLC-MS platform utilizing a novel monolithic RP capillary column for TDP of an *E. coli* lysate, identifying 347 proteoforms larger than 30 kDa in a single RPLC run.^28^ While advanced SEC and RPLC techniques have been widely utilized to identify large proteoforms, the global characterization of large proteoforms from cell lysates remains challenging. One of the primary reasons is the limited separation capacity of SEC and RPLC for large proteoforms.

**Fig. 2:**
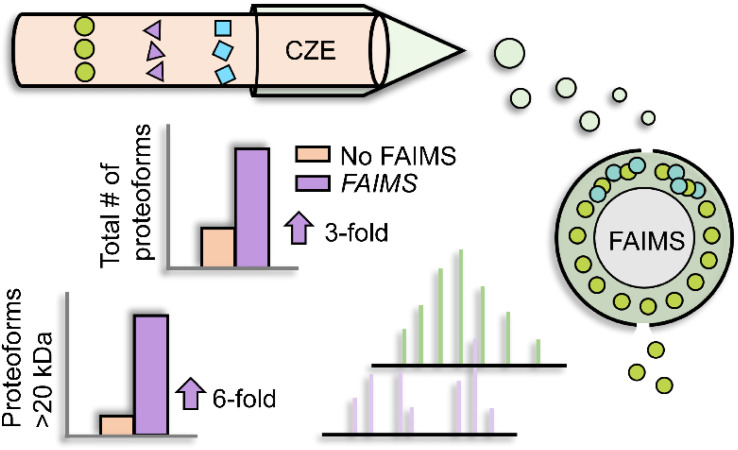
Schematic of capillary zone electrophoresis (CZE)-field asymmetric waveform ion mobility spectrometry (FAIMS)-mass spectrometry (MS) and MS/MS for top-down proteomics (TDP) of a complex sample. Using nine different compensation voltages, FAIMS achieved online gas phase fractionation, resulting in a nearly 3-fold increase in the number of proteoform IDs. This method particularly benefited the identification of relatively large proteoforms compared to CZE-MS/MS alone. Reproduced from ref. 40 with permission from the American Chemical Society, copyright 2023.

Besides LC, electrophoretic methods are promising for large proteoform separations. Capillary zone electrophoresis (CZE) separates analytes under an electric field according to their charge-to-size ratios. As CZE performs separation in an open tubular fused silica capillary containing no stationary phase, it can achieve higher separation efficiency and less sample loss for large proteoforms than many conventional LC techniques.^29–33^ Additionally, CZE-MS/MS has been well recognized as a valuable technique for TDP as the nanoflow CE-MS interface (*i.e.*, 50–300 nL min^−1^) provides high sensitivity for detection.^32–38^ The Kelleher group utilized a GELFrEE (gel-eluted liquid fraction entrapment electrophoresis)-CZE-MS/MS platform to improve the detection of large proteoforms.^39^ Thirty proteins in the mass range of 30–80 kDa from the *Pseudomonas aeruginosa* whole-cell lysate were identified. The Sun group coupled FAIMS (high field asymmetric waveform ion mobility spectrometry) to CZE-MS/MS to achieve online two-dimensional separations (IMS and CZE) for improving the ID of large proteoforms, Fig. 2.^40^ By altering the compensation voltages from −50 to 30 V, FAIMS enabled the efficient fractionation of proteoform ions in the gas phase, with a median proteoform mass range from less than 10 kDa to about 30 kDa. CZE-FAIMS-MS/MS boosted the number of proteoform IDs six-fold in the 20–45 kDa range. Apart from CZE, capillary isoelectric focusing (cIEF) has been well known for ultra-high resolution protein separation based on their isoelectric points (pIs).^41,42^ cIEF-MS has been widely used for top-down MS characterization of proteoforms.^32,43^ Several research groups have demonstrated that cIEF-MS is highly efficient for the characterization of charge variants of monoclonal antibodies (mAbs, ∼150 kDa).^43–47^ Recently, the Sun group developed a native cIEF-MS technique for the delineation of large protein complexes and the accurate determination of pIs of various forms of large protein complexes.^48^ More studies are still needed to improve the performance of CE-MS/MS for the global characterization of large proteoforms from cell lysates. The Takemori group developed passive eluting proteins from polyacrylamide gels as intact species for MS (“PEPPI-MS”) for MS-intensive TDP.^49^ The PEPPI is a gel-based electrophoresis method for high-resolution separation of proteoforms based on their masses and recovery of proteoforms from polyacrylamide gels. A 68% median recovery rate for <100 kDa proteins and a 57% median recovery rate for proteins larger than 100 kDa were reported previously. Coupling PEPPI to RPLC-MS and MS/MS identified nearly 60 proteoforms higher than 30 kDa from an *E. coli* cell lysate. Although PEPPI efficiently separates large proteoforms, the PEPPI workflow includes protein cleanup steps before MS analysis. More careful evaluations are needed for comprehensive proteoform ID and accurate quantification using the PEPPI-based approach.^50^

Significant efforts have also been made to improve the MS instrumentation to enable accurate mass determination of large proteoforms and to boost the sensitivity of TDP for large proteoforms. One big issue of large proteoform ID is the unresolved isotopic peaks in each charge state due to the limited mass resolution of mass spectrometers, making accurate charge determination challenging.^17,23^ The development of charge detection MS (CDMS) provided a useful solution for large proteoforms, enabling mass analysis of megadalton particles with the direct readout of an ion's charge.^51^ At the single-ion regime, the measured image current is proportional to the charge of the ion, enabling CDMS.^52^ Orbitrap-based individual ion MS (I2MS) determines the charge of individual ions by acquiring hundreds of mass spectra with a single ion per scan.^53^ It then creates high-resolution mass spectra of proteoforms and protein complexes (megadalton) for accurate mass determination. The I2MS technique substantially boosts TDP's sensitivity compared to conventional data acquisition with an ensemble of ions. I2MS detected over 500 proteoforms from a GELFrEE fraction of a human cell lysate *via* direct sample infusion, and the conventional data acquisition failed to detect any clear proteoform signal. Further coupling I2MS with ambient nanospray desorption electrospray ionization (nanoDESI) enabled proteoform-level imaging of tissue samples with the detection and ID of hundreds of proteoforms up to 70 kDa.^54^ The Heck group also developed a novel single-molecule MS technique using an Orbitrap mass analyzer through frequency chasing of individual megadalton ions.^55,56^ The technique has reached incredibly high mass resolution (>100 000) at *m*/*z* 35 000 and enabled accurate mass determination of megadalton complexes. Coupling these single-ion MS techniques with in-front high-capacity liquid-phase separations will drastically advance the TDP of large proteoforms and protein complexes.

Another big challenge of large proteoform ID with the TDP approach is the limited backbone cleavage coverage from conventional gas-phase fragmentation techniques (*i.e.*, collision-induced dissociation, CID, and higher energy collision dissociation, HCD), impeding the accurate localization of PTMs and sequence variations. Electron or photon-based fragmentation techniques (*i.e.*, electron transfer dissociation, ETD, electron capture dissociation, ECD, and ultraviolet photodissociation, UVPD) have demonstrated their substantially better performance than HCD for large proteoform fragmentation.^57–59^ However, even with advanced fragmentation methods, resolving the overlapping peaks and complex spectra of large proteoforms remains challenging. The spectral complexity introduced by multiple charge states can be reduced by ion–ion reactions, such as proton transfer charge reaction (PTCR), where a multiply charged cation reacts with a singly charged reagent anion to generate cations with reduced charge.^60^ For intact proteoforms, using PTCR with isolated precursors (<1.5 *m*/*z*) has doubled the identification of proteoforms over 30 kDa.^61^ Broadband isolation of the entire charge state distribution of intact proteins enabled the detection of proteins ranging from 14 to 148 kDa.^62^ At the tandem mass spectra scale, PTCR limited signal overlap produced by ETD and improved the sequence coverage of proteins spanning from 29 to 56 kDa.^63^ The PTCR MS^3^*via* HCD further increased the sequence coverage of 56 kDa glutamate dehydrogenase to 44%. Post-activation ion mobility spectrometry also simplified the mass spectra and enabled unambiguous assignment of typically overlapping product ions.^64^

### 2.2 MS-intensive TDP of membrane proteins

Despite membrane protein-coding genes being projected to constitute around 30% of the human proteome, membrane proteins (MPs), which play critical roles in cellular function and serve as therapeutic targets, tend to be underrepresented in most global proteomic studies. The low solubility of MPs in MS-compatible buffers is the main issue of MS-based TDP of MPs. A typical top-down MS analysis of membrane proteoforms requires detergent micelles, organic solvent, ionic liquid, or membrane mimetics (*e.g.*, nanodiscs, lipid vesicles, bicelles) to keep the solubility prior to MS.^65–68^ Great efforts have been made in extraction methods, separation techniques, and MS instrumentation during the last several years to advance TDP of MPs.

Advanced MP purification/extraction methods with multidimensional liquid-phase separations have been used for MP characterization by TDP. The Ge group developed a novel photo-cleavable surfactant (Azo) to enable TDP of integral membrane proteins (IMPs) by providing the solubility of IMPs and compatibility with MS using UV irradiation-based degradation.^69^ More work remains to be done to make Azo more broadly used in TDP studies, *e.g.*, more systematic and critical evaluations of Azo for IMPs in terms of degradation, MS interference, and its compatibility with online LC-MS/MS or CE-MS/MS. They also used cloud point extraction to enrich the MPs and identified 188 and 124 endogenous IMPs from human embryonic kidney cells and cardiac tissues, respectively, *via* coupling SEC with RPLC-MS/MS.^70^ The Zhang group developed a novel C8-functional amine-bridged hybrid monolith capillary column for RPLC-MS/MS analysis of MPs.^71^ Integrating ultracentrifugation-based MP purification, GELFrEE fractionation, and RPLC-MS/MS identified 3100 membrane proteoforms up to 50 kDa from a mouse hippocampus sample, representing the largest TDP dataset of membrane proteoforms. Beyond RPLC-MS/MS, CZE-MS/MS has also been deployed for the TDP of MPs. The Sun group recently developed an efficient CZE-MS/MS method for TDP of IMPs from a mouse brain sample.^72^ The method employs a sample buffer containing 30% (v/v) formic acid and 60% (v/v) methanol for solubilizing IMPs and utilizes a separation buffer of 30% (v/v) acetic acid and 30% (v/v) methanol for maintaining the solubility of IMPs during the CZE separation. Coupling SEC fractionation with CZE-MS/MS identified 276 IMP proteoforms with 1–4 transmembrane domains (TMDs), demonstrating the high potential of SEC-CZE-MS/MS for TDP of IMPs. However, further improvements are required for the global characterization of large IMPs using CZE-MS/MS.

Advancements in MS instrumentation have allowed more accurate characterization of MPs and MP complexes. The Robinson group delineated rhodopsin receptor signaling cascade in a native membrane using an advanced Orbitrap mass spectrometer.^73^ Various gas-phase fragmentation techniques (*i.e.*, ETD, CID, HCD, UVPD, and infrared multiphoton dissociation, IRMPD) have been validated for IMP fragmentation. ETD favors the cleavage in the soluble region of IMPs, while CID and HCD have a dramatic bias towards TMDs, due to the lack of basic residues.^74^ Compared with the 28% sequence coverage of the c-subunit of ATP synthase (8 kDa) by CAD, activated ion ECD (ai-ECD) achieved 72% coverage by irradiating the precursor ion using infrared prior to ECD.^75^ Regarding the fragmentation of MP complexes from native MS, the sequence coverage is typically relatively low (10–20%).^76^ UVPD fragments protein ions *via* the absorption of high-energy photons. The ions are directly dissociated without energy redistribution, preserving bound ligands and labile PTMs.^77^ UVPD enhanced the sequence coverage of aquaporin Z (AqpZ, 99 kDa) and mechanosensitive channel of large conductance (MscL, 79 kDa) to 45% and 53%, respectively, compared with 30% and 34% obtained from HCD.^78^ However, the enhancement of UVPD is also highly dependent on the membrane complex structure and sequence. Infrared multiphoton dissociation (IRMPD) accumulates many low-energy photons and gradually increases the vibrational energy to break the amide (C–N) bonds. It has also been used to liberate IMPs from micelles.^79^ Using a modified Orbitrap Eclipse Tribrid MS with a continuous wave far IR CO_2_ laser, the Robinson group achieved selective detergent removal and a successive cleavage of adjacent residues in TMDs with a 26% sequence coverage of AmtB.^80^ Moreover, the Ruotolo group improved IMP sequence coverage to 40–60% by applying IR photoactivation before HCD.^81^ While significant progress has been made in MS instrumentation for more comprehensive characterization of MPs, combining advanced MP extraction and separation techniques with novel MS/MS methodologies for global characterization of MPs in whole cell lysates has not been done and needs further effort.

### 2.3 MS-intensive TDP for global profiling of proteoforms in complex samples

Global TDP profiling of complex samples is impeded by the extremely high sample complexity. Multi-dimensional separations of proteoforms before MS and MS/MS have been utilized to boost the proteome coverage of MS-based TDP.

The Kelleher group applied cell selection by immunomagnetic enrichment and fluorescence-activated cell sorting (FACS) to selectively enrich specific cell types from human blood samples, followed by RPLC-MS/MS-based proteoform profiling.^82^ Nearly 30 000 nonredundant proteoforms and 1690 proteins were identified from 21 different human hematopoietic cell types and plasma. Thousands of proteoforms were identified from each cell type. The Kelleher group also combined CZE-MS/MS and RPLC-MS/MS to characterize proteoforms across five human tissues with an ID of ∼11 500 proteoforms.^12^ They showed that proteoforms from CZE and RPLC approaches are complementary, and CZE-MS/MS offers a much better sensitivity than RPLC-MS/MS for proteoform IDs. The Sun group coupled LC fractionation to CZE-MS/MS for TDP of colorectal cancer cells (CRC cells, SW480 and SW620) and identified 23 622 proteoforms of 2332 proteins.^5^ The technique improved the number of proteoform IDs by nearly fivefold compared to previous TDP datasets of human cancer cells. In addition, the Sun group coupled SEC to automated cIEF-MS/MS for TDP of zebrafish brains with the ID and quantification of thousands of proteoforms.^83^ The Wu group developed an online two-dimensional ultrahigh-pressure nano-LC system for high-pH and low-pH RPLC separations of proteoforms prior to MS and MS/MS.^84^ The online platform identified over 1000 intact proteoforms from only 5 μg of an *E. coli* cell lysate. Those studies document that high-capacity liquid-phase separations (LC and CE) are vital for global proteoform profiling from cells and tissues. Dramatic improvement in proteoform separation is still needed to characterize proteoforms in cells or tissues comprehensively.

**Fig. 3:**
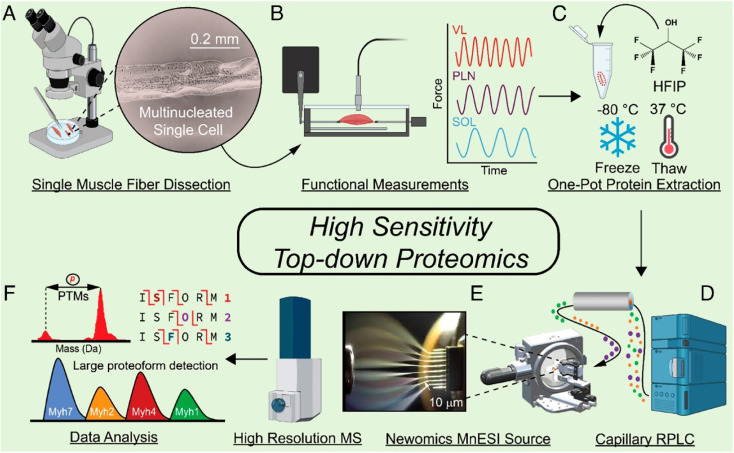
Schematic of the highly sensitive top-down proteomics (TDP) workflow for the characterization of proteoforms in single muscle fibers, including the mechanical dissociation of SMFs (A), velocity measurement of each muscle in a force transducer (B), protein extraction (C), capillary RPLC separation of SMF extracts (D), MnESI ionization of SMF proteins coupled with high-resolution MS analysis (E), and data analysis of the TDP data by MS/MS as well as the large proteoform detection (F). Reproduced from ref. 27 with permission from the National Academy of Sciences (NAS), copyright 2023.

With the improvement of the sample preparation method and LC-MS/MS or CE-MS/MS, achieving TDP measurement of proteoforms in mass-limited biological samples (*i.e.*, nanoproteomics in single cell scale) is possible. Nanoproteomics refers to the proteomics analysis of small cell populations, spanning from individual cells to clusters of cells that contain less than 1 μg protein amount in total.^85,86^ Coupling nanoPOTS-based sample preparation^87^ and advanced capillary RPLC-MS/MS has enabled TDP of a small number of mammalian cells with the ID of hundreds of proteoforms from tens to hundreds of HeLa cells.^88^ The Ge group developed a highly sensitive TDP workflow for SMFs, Fig. 3.^27^ They employed a one-pot sample processing approach to reduce protein loss and a capillary RPLC-MS/MS system with micro-flow-nanospray ionization (MnESI) to boost the sensitivity of MS measurement. They observed significant phosphorylation heterogeneity of proteoforms of myosin light chain 2 (MLC-2) in different SMFs. The Ivanov group reported TDP of single mammalian cells using CZE-MS/MS with the assistance of on-capillary cell lysis.^89^ Dozens of proteoforms were identified by MS/MS from single HeLa cells. The Sun group integrated laser capture microdissection, one-step sample processing, and CZE-MS/MS for TDP of isolated zebrafish brain regions.^11^ Over 400 proteoforms were identified from one LCM brain section with a 500 μm^2^ area and 20 μm thickness. Recently, the Wu group developed a novel CZE-MS platform (Spray capillary) for handling pL–nL level biological samples for CZE-MS analysis.^90^ Although the technique has not been thoroughly investigated for TDP of small numbers of mammalian cells, it showed exciting results for single-cell analysis of metabolites. These technical advancements open the door to MS-based TDP of mass-limited biological samples. However, substantial work is still needed to improve the whole TDP workflow for reliable and quantitative measurement of proteoforms in trace samples.

### 2.4 MS-intensive TDP of highly modified proteoforms

Proteoforms can be heavily modified by various PTMs such as phosphorylation, methylation, acetylation, and glycosylation. The PTMs regulate protein functions. Delineation of proteoforms carrying various PTMs (*e.g.*, histone) is vital for understanding their biological functions and is a challenging task for MS-based TDP.

Histones, acting as the fundamental framework within the nucleosome where genomic DNA is intricately packed, are essential for both the regulation of epigenetic gene expression and the maintenance of chromatin's structural stability^91–95^ The protruded N-terminal tails of the four core histones (H2A, H2B, H3, and H4) from the nucleosome structure contain a wide variety of PTMs, such as methylation, acetylation, phosphorylation, and citrullination.^93,96^ With the diverse PTMs and high heterogeneity of histones, fully deciphering how histone proteoforms contribute to epigenetic gene regulation remains difficult.^97,98^ High-resolution separation and extensive gas-phase fragmentation of histone proteoforms are crucial. The Sun group developed SEC-CZE-MS/MS (HCD) and CZE-FAIMS-MS/MS (HCD) for high-capacity separation and highly sensitive TDP measurement of histone proteoforms, leading to the ID of nearly 400 and 600 histone proteoforms from a commercial calf thymus sample.^99,100^ PTM localization for TDP analysis continues to pose a hindrance because the production of substantial fragment ions from highly efficient gas-phase fragmentation is vital for confidently mapping the sequence and confirming the PTM information.^101^ To accurately localize the PTMs on histone proteoforms, the Brodbelt group integrated UVPD with gas-phase PTCR to achieve up to 91% sequence coverage of calf thymus histone H4 with N-terminal acetylation, K12 acetylation, and R3 dimethylation.^102^ To separate highly isomeric histone proteoforms, the Jensen group applied trapped ion mobility spectrometry (TIMS)-time-of-flight (TOF) to resolve the isomers of monoacetylated H4 histone, providing a useful approach for the separation of highly similar histone proteoforms by their conformational differences.^103^ Another challenge for TDP analysis of histone proteins is associated with accurately differentiating the near-isobaric modifications, such as distinguishing acetylation and trimethylation as well as formylation and dimethylation (Δ*M* = 0.0364 Da). The high mass resolution MS/MS and the low mass tolerance are extremely essential to assigning correct fragment ions of histone proteoforms.^104^ We expect that researchers will integrate the advanced separation techniques, gas-phase fragmentation methods and high-resolution mass spectrometers together soon to characterize histone proteoforms better.

Protein glycosylation modulates a wide range of cell functions and is a highly diverse type of PTM.^105,106^*N*- and *O*-linked glycosylation are the two main types of glycosylation. Due to the enormous structural microheterogeneity, efficient separation and ultra-high-resolution MS measurement are necessary for the top-down MS characterization of glycoproteoforms. The spike (S) protein, essential for severe acute respiratory syndrome coronavirus 2 (SARS-CoV-2) virus entry, is highly glycosylated and contains 66 potential *N*-linked glycosylation sites within each trimer.^107–109^ Interestingly, three possible *O*-linked glycosylation sites have been predicted, and two of them were identified at the receptor-binding domain (RBD) of the S protein subunit S1.^110,111^ The abundant glycosylation on S protein enables the SARS-CoV-2 to escape innate and adaptive immune responses by protecting the epitopes from antibody neutralization.^109,112^ However, due to the enormous microheterogeneity of *N*-glycans and *O*-glycans, each glycosylation site could result in numerous possibilities of variable glycans with different arrangements and branched structures. Therefore, interpreting highly glycosylated S protein in a proteoform-specific manner is essential to provide new insights into virus binding, fusion, cell entry, and the development of vaccines. The Ge group explored the structural heterogeneity of different conformers of S protein RBD by native top-down TIMS-MS/MS and applied ultrahigh-resolution Fourier transform ion cyclotron resonance (FTICR)-MS/MS to deeply analyze the glycosylation sites and the corresponding glycans by denatured top-down MS.^113^ Overall, they disclosed eight S protein RBD *O*-glycosylated proteoforms, a core 2-fucosylated glycan structure, and the relative quantification information, proving that the combination of IMS and ultrahigh-resolution MS is capable of characterizing highly glycosylated S protein RBD. Another approach to investigate the glycosylation heterogeneity of S protein is using CDMS. The Jarrold group utilized top-down CDMS measurements to study the heterogeneity of glycosylation at the 66 *N*-glycan sites of S protein trimer, offering much more accurate insights into the glycosylation heterogeneity of S protein compared to bottom-up glycoproteomics data.^114^ Those works studied purified glycoproteins using the top-down MS approach. However, much effort must be made to achieve global profiling of glycoproteoforms in cells or tissues.

### 2.5 MS-intensive TDP for high-throughput proteoform quantification

Most quantitative TDP studies employ a label-free approach for relative proteoform quantification by comparing the proteoform intensity across different biological conditions.^5,82,115,116^ The label-free approach is simple and applicable to various biological samples. However, when analyzing many biological samples, it suffers from low throughput due to the extended time required for multiple sample preparations, effective online separation, high-resolution mass spectrometry, and time-consuming data processing. Different stable isotopic labeling methods have been investigated to boost the throughput of MS-based quantitative TDP.^117–125^ Tandem mass tags (TMT) and dimethyl isotope labeling have recently been systematically studied for quantitative TDP of complex cell lysates.^121–125^ The Wang group developed a dimethyl isotope labeling approach for LC-MS/MS-based quantitative TDP and quantified over 2000 proteoforms from yeast cell lysates under anaerobic and aerobic conditions.^121^ The isotopic labeling efficiency can be as high as 99%. The Wu group developed TMT-based approach for quantitative TDP of complex samples^122–125^ and quantified hundreds of proteoforms from *E. coli* cell lysates.^122^ The TMT labeling efficiency for *E. coli* proteoforms was better than 80%. Most recently, the Wu group developed an optimized workflow for TMT-based quantitative TDP and applied the approach to HeLa cell lysates, enabling the quantification of hundreds of proteoforms per LC-MS/MS run with the TMT labeling efficiency better than 90%.^125^ The results indicate that the dimethyl and TMT labeling approaches are promising for high-throughput quantitative TDP of complex samples. However, some issues still exist for those strategies. First, labeling efficiency depends on many factors, for example, the structure and size of the proteoforms. It is possible to have under-labeling and over-labeling situations, which will undoubtedly impact the proteoform ID and quantification. Second, it is challenging to label large proteoforms using, for example, TMT, due to the high organic solvent concentration in the labeling system, which leads to the precipitation of large proteoforms.^122^ Third, we need to balance the quality of proteoform ID and quantification for the TMT-labelled proteoforms because fragmentation of TMT labeling and proteoform backbone requires drastically different energies.^125^ Finally, advanced bioinformatics tools are needed to process the data for relative proteoform quantification based on the stable isotopic labeling approaches. The most widely used bioinformatics tools for MS-based TDP usually mainly support label-free-based proteoform quantification.

### 2.6 Bioinformatics tools for MS-intensive TDP

Advanced bioinformatics tools are extremely important for the accurate identification and quantification of proteoforms in MS-based TDP. Two main approaches for TDP analysis is either by searching against an annotated database such as ProSight, which uses Poisson matching for the reported modifications in the UniProtKB XML,^15^ or against a protein sequence database like TopPIC suite, which employs spectral alignment based on mass ladders and nonspecific mass shifts.^14^ Notable tools include but not limited to pTop – a spectral alignment software incorporating machine learning for detecting precursors more precisely,^126^ Informed Proteomics – a spectral alignment suite using a graph-based approach combining with LC-MS feature,^127^ MetaMorpheus – integrating database search of BUP and TDP,^128^ and MASH – an interface of multiple algorithms and visualization.^16^ In the last several years, some important advancements in bioinformatics tools have improved proteoform characterization in MS-based TDP studies.

The current bioinformatics tools for MS-based TDP have unsatisfied performance for ID of large proteoforms (*i.e.*, >30 kDa) because the software typically relies on isotopically resolved peaks in each charge state to determine the accurate monoisotopic mass of proteoforms or their fragment ions for subsequent database searches, which is called deconvolution. Typical high-resolution mass spectrometers cannot offer high enough mass resolution to achieve isotopically resolved peaks for large proteoforms. Alternatively, for unresolved isotopic peaks, the average mass can be determined based on the charge state distribution, which is utilized in UniDec and TDPortal.^129^ Minor changes in deconvolution parameters may resolve distinct results, and the erroneous deconvolution of proteoforms leads to incorrect false discovery rate estimation at the proteoform-level.^130^

Recently, Jeong *et al.* presented an ultrafast deconvolution tool for TDP, FLASHDeconv, capable of analyzing both isotopically resolved and unresolved peaks for broad charge and mass range in MS spectra, which is especially useful for large proteoform ID.^131^ The same research group further developed an intelligent online data acquisition algorithm FLASHIda, enabling ultrafast proteoform deconvolution in real time during data acquisition.^132^ The commonly used data-dependent acquisition (DDA) method selects the top N most intense peaks for fragmentation. However, this method tends to choose multiple charge state peaks from a high abundance proteoform, leading to the difficulty in the ID of low abundance proteoforms. FLASHIda enables less redundant selection of precursor ions *via* real-time FLASHDeconv, boosting the proteoform coverage in MS-based TDP studies. There is another real-time deconvolution and precursor selection software for MS-based TDP, MetaDrive, which aims to combine the multiple charge states of the same proteoform to improve the quality of the MS/MS spectra and the backbone cleavage coverage of proteoforms.^133^ This approach could be potentially valuable for bettering the sequence coverage of large proteoforms.

Another reason of complex spectra is the internal fragment ions, which contain neither N- nor C-terminus of proteoforms and are not recognized by most software, especially when a combination of fragmentation is employed.^134,135^ More recently, the Loo group highlighted the value of internal fragment ions for boosting proteoform sequence coverage from various fragmentation techniques and developed a bioinformatics tool (*i.e.*, ClipsMS) to facilitate the use of internal fragment ions in top-down MS studies.^136–140^ The information on internal fragment ions improves the sequence coverage of large proteoforms substantially. The Loo group combined ECD (e-MSion), HCD, and internal fragment ion assignments for top-down MS analysis of the whole NIST mAb, producing a 75% sequence coverage, the highest sequence coverage of an intact mAb by TDP so far.^139^ Besides the ClipsMS software by the Loo group, the Smith group implemented internal fragment ion functionality in their MetaMorpheus software for MS-based TDP of cell lysates with internal fragment ion annotation.^141^ The approach improved the number of proteoform IDs and the distinguishment of similar proteoforms significantly compared to the conventional terminal fragment ion approach. However, the internal fragment ion assignment requires high mass accuracy and special attention in controlling false positive hits. Additionally, to make the internal fragment ions more useful for general MS-intensive TDP studies, integration of automated annotation of internal fragment ions in the most widely used bioinformatics tools (*i.e.*, ProSight^15^ and TopPIC^14^) will be critically important.

Although various bioinformatics tools have been developed to improve the quality of proteoform characterization, alternative methods are still needed to verify the identified proteoforms from the database search, especially those proteoforms carrying PTMs (*e.g.*, phosphorylation and acetylation). The Sun group reported high linear correlations (*R*^2^ = 0.98) between predicted and experimental electrophoretic mobility of proteoforms identified by CZE-MS/MS-based TDP.^142^ The Liu group also demonstrated the solid linear correlations between the predicted and experimental migration/retention time of proteoforms identified in CZE-MS/MS and RPLC-MS/MS studies.^143^ More importantly, the proteoforms carrying phosphorylation and/or acetylation PTM can be confirmed by comparing their predicted and experimental electrophoretic mobility due to the charge reduction from those PTMs.^142^ The results indicate that integrating the predicted and experimental electrophoretic mobility or retention time information into the database search bioinformatics tools could potentially improve the confidence and accuracy of proteoform IDs. The Liu group also developed a new bioinformatics tool, PTM-TBA (PTM characterization by top-down and bottom-up MS and annotations), based on the TopPIC software to better the localization of PTMs on identified proteoforms *via* combining the top-down and bottom-up MS data and PTM annotations in the UniProt database.^144^

Besides proteoform ID, some new bioinformatics tools have been developed for label-free proteoform quantification. The Tholey group developed MSTopDiff to improve the annotation of artificial and biological modifications of the detected proteoforms.^145^ The Petyuk group developed a companion R package (TopPICR) for TopPIC to enable label-free quantification.^146^ The TopPICR tool performs many functions related to label-free quantification, including ID filter, retention time alignment across runs, mass recalibration, and proteoform feature intensity calculation with the match-between-runs approach.

One significant problem in bioinformatics for MS-based TDP is that proteoform IDs from the same dataset using different bioinformatics tools are substantially different in deconvolution results and proteoform IDs.^147^ This phenomenon might be due to the differences in, for example, mass deconvolution, the database for search, and the scoring algorithm for matching experimental and theoretical data for the TDP bioinformatics tools. The Ge group built MASH Explorer as a universal and user-friendly software for MS-based TDP.^148^ The software combines several widely used spectral deconvolution tools and database search algorithms into one platform, providing flexibility and consistency for proteoform IDs using TDP. It can also process TDP data from different vendor formats, which is particularly useful for the broad TDP community.

## 3. Biomedical applications of MS-intensive TDP

In recent years, MS-based TDP has been applied to various biomedical applications due to substantial technological improvement. The studies focus on mapping the proteoform landscape of disease-relevant samples or disease-specific genes for a clear understanding of the molecular mechanism of disease development, the discovery of potential biomarkers for disease diagnosis, and the innovation of precise therapeutics.

### 3.1 Cancer biology

**Fig. 4:**
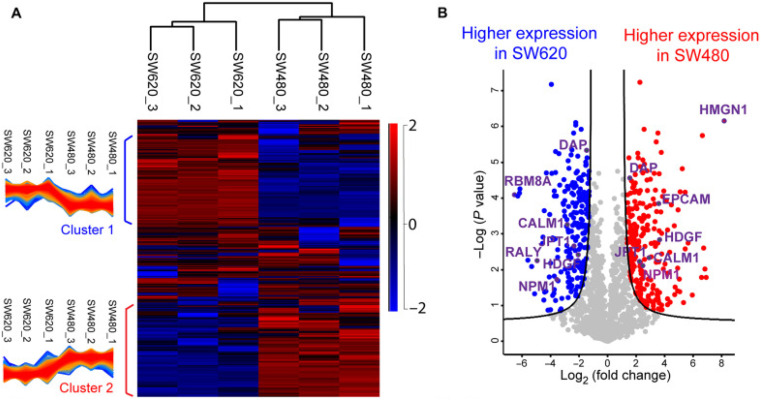
Summary of the label-free proteoform quantification data of metastatic (SW620) and non-metastatic (SW480) colorectal cancer (CRC) cells from size exclusion chromatography (SEC)-capillary zone electrophoresis (CZE)-tandem mass spectrometry (MS/MS). (A) Heatmap and clusters of the quantified proteoforms from the two cell lines. (B) Volcano plot analysis indicating significantly differentially expressed proteoforms. Reproduced from ref. 5 with permission from the American Association for the Advancement of Science (AAAS), copyright 2022.

Proteomics profiling of protein changes in the cancer process benefits cancer biomarker discovery and understanding of cancer-specific mechanisms and phenotypes. BUP was the dominant proteomic approach in cancer research in the past decades but is challenging in capturing the combinatorial PTMs or sequence variations in proteins.^149–151^ Rapid dissemination of TDP is filling this gap. The Kelleher group integrated TDP and BUP to study patient-derived breast tumor xenografts and demonstrated the significance of TDP in acquiring aberrations of cancers at the proteoform level.^152^ The Sun group performed a comprehensive TDP study of a pair of metastatic and nonmetastatic CRC cell lines (SW480 and SW620 cells).^5^ It revealed the drastic transformation of CRC cells in proteoform profiles after metastasis, Fig. 4. The study disclosed differentially expressed phosphorylated proteoforms of important cancer-related genes in well-known CRC pathways between the SW480 and SW620 cell lines, and those proteoforms could be proteoform biomarkers of CRC metastasis. Targeted immunoprecipitation (IP)-TDP characterization and quantification of KRAS4b proteoforms in CRC cell lines and tumors by the Kelleher group revealed crosstalk between genetically encoded mutations and proteoform PTMs.^153^ Their follow-up TDP study of KRAS isoforms (KRAS4a and KRAS4b) across various cell and tissue samples showed distinct modifications between the two isoforms.^7^ Functional characterization of a KRAS4b proteoform with truncation at C185 residue revealed their loss of the ability to interact with the plasma membrane, resulting in decreased activation of downstream MAPK signaling. Most recently, D'Ippolito *et al.* developed an LC-MS/MS-based TDP approach to assess KRAS4B compound engagement *in vitro*, which is promising to be applied for wide screening of targeted KARS inhibitors for cancer treatment.^154^ Holt *et al.* presented a quantitative TDP method with high throughput for studying triple-negative breast cancer cells. It was typically used to evaluate an HDAC inhibitor's impact on histone H4 proteoforms to probe chromatin dynamics and interactions.^155^ McGee *et al.* innovated proteoform imaging by developing an individual-ion MS-based multiplexed workflow for top-down tandem MS and label-free quantification. Out of around 1000 identified proteoforms from the same patient, 303 differential proteoforms were discovered between stroma and tumor.^156^ We expect more and more MS-based TDP studies to be performed in either discovery or targeted mode to bridge cancer genotypes and phenotypes and assist cancer therapeutics development.

### 3.2 Neurodegenerative diseases

Several key proteins, such as amyloid beta, alpha-synuclein, tau, *etc.*, are closely involved in the pathogenesis of Alzheimer's disease (AD), Parkinson's disease (PD), and amyotrophic lateral sclerosis (ALS). The Petyuk group developed a RPLC-FAIMS-MS/MS technique for TDP of brain tissue from an AD patient and achieved the identification of a variety of proteoforms from synuclein, tau, and amyloid beta.^157^ In particular, diverse fragments of splice isoforms of tau found in this study provide insight into their proteolytic degradation pathways and their functions. The Knierman group characterized 11 alpha-synuclein proteoforms with truncations or PTMs (*e.g.*, phosphorylation) across Parkinson's disease tissues and normal brain tissues.^158^ The proteoform carrying serine-129 phosphorylation, a widely accepted pathology-associated modification, presented significant differences between disease tissues and controls. The Agar group coupled hydrogen–deuterium exchange with MS to investigate the structural perturbation of SOD1 induced by mutations and PTMs.^159^ They observed that diverse mutations and PTMs of SOD1 could cause its structural perturbation, which is potentially associated with the progression of sporadic ALS. The DeMarco group recently introduced their work using targeted qualitative and quantitative MS to discover frontotemporal lobar degeneration (FTLD)-specific TDP-43 proteoforms.^160^ They found truncated TDP-43 proteoforms, especially C-terminal fragments, were highly enriched in FTLD-TDP cases, which can serve as a biomarker with high diagnostic accuracy. Those studies have demonstrated the potential of MS-based TDP for providing novel and useful proteoform-specific information about neurodegenerative diseases. However, MS-based TDP of vital neurodegenerative disease-related proteins, *e.g.*, tau, faces some technical challenges. Intact tau is heavily modified and large (>30 kDa).^161^ Loo lab implemented direct spray-based native TDMS with ECD fragmentation and ion mobility MS to explore PTMs and structural information of recombinant tau and characterize CLR01 inhibitor binding site on tau.^162^ Extensive TDP measurement of complex tau proteoforms in endogenous samples still requires high-capacity LC/CE separation, highly sensitive MS detection, and extensive gas-phase fragmentation technologies.

### 3.3 Cardiac injury

Cardiac injury causes millions of deaths worldwide each year.^163^ The underlying molecular mechanism of cardiac injury and dysfunction remains unclear. Cardiac troponin I (cTnI) is a key cardiac regulatory protein and a biomarker of cardiac injury. It can form complexes with other cardiac troponin (cTn) proteins to modulate calcium-mediated interaction between actin and myosin to control cardiac contraction. The Ge group performed immunoaffinity enrichment and quantitative TDP of cTnI in normal and diseased heart tissues and reported a strong correlation between phosphorylation on Ser22/23 and heart disease phenotype.^164^ They also developed a novel nanoproteomics approach based on surface functionalized nanoparticles for sensitive enrichment and TDP characterization of cTnI proteoforms from serum, which is highly applicable for disease diagnosis and evaluation of treatment progress.^165^ Moreover, their TDP studies were also extended to define sarcomeric proteoform landscape underlying hypertrophic cardiomyopathy (HCM) and ischemic cardiomyopathy (ICM) pathophysiology.^166,167^ In HCM cases, the concerted reducing phosphorylation in crucial myofilament and Z-disk proteins manifested their PTM crosstalk in the sarcomere and dysregulation of protein kinase A pathways.^166^ Altered sarcomeric proteoforms were also observed in HCM patients receiving septal myectomy, which are aligned with the same clinical phenotypes despite their differences in HCM-induced mutations. Quantitative TDP comparison of nonfailing donors and end-stage ICM patients showed differential expressions of sarcomeric proteins (*e.g.*, cTnI, ENH2, β-Tpm, α-SKA, *etc.*) and modifications (*e.g.*, phosphorylation increase in cTnT, calsarcin-1).^167^ Additionally, Bodin *et al.* combined TDP with BUP and MALDI-TOF MS to profile acute pathophysiological myocardial changes after cardiac electrical shocks using a sheep model.^168^ Ramirez-Sagredo *et al.* applied quantitative TDP to study alternation of intact mitochondrial proteoforms in murine hearts associated with cardiac aging.^169^ The data in those studies demonstrate the clinical value of proteoform-specific measurement using MS-based TDP.

### 3.4 Diabetes and cardiovascular diseases (CVD)

Apolipoproteins are important components of lipoprotein particles for transporting blood lipids and are closely associated with diabetes and cardiovascular disease. Apolipoproteins A-I (APOA1, 243 amino acids) and A-II (APOA2, 77 amino acids) are the two most abundant proteins in high-density lipoproteins (HDLs) particles that participate in lipid metabolism.^170^ The Wang group developed a novel microfluidic-chip-based nanoLC-MS platform for direct monitoring of crucial diabetes biomarkers, including glycated and oxidated APOA1.^171^ Another TDP study of apolipoproteins reported typical higher abundant APOA1 and APOA2 oxidation in patients with type II diabetes (T2D) and CVD.^170^ A recent targeted TDP study combining selected-ion monitoring and ETD MS characterized eighteen AOPA1 proteoforms in human serum. The quantitative comparison of two groups of participants with the highest and lowest HDL cholesterol efflux (HDL-E) highlighted elevated canonical, carboxymethylated, and fatty acylated APOA1 proteoforms in high HDL-E patients.^172^ The Kelleher group introduced a novel procedure coupling native GELFrEE with LC-MS to study APOA1 and APOA2 proteoform heterogeneity across different HDL particle sizes.^173^ The work indicated higher acylated (fatty acid modification) APOA1 levels in larger HDL particles but similar APOA2 proteoform abundance across size fractions. Besides APOA1 and APOA2, Apolipoprotein C-III (APOC3, 79 amino acids) is also associated with coronary artery disease. APOC3 presents in both HDL and low-density lipoproteins (LDL) and functions as a regulator to control plasma triglyceride levels.^170,174^ Quantitative TDP of apolipoproteins in human HDL by Mazur *et al.* found specific APOC3 glycoform linked to coronary artery disease.^175^ TDP of APOC3 detected various glycoforms that share a core glycan chain but with variations in sialic acid residues.^170^ The Nedelkov group reported significantly greater ratios of non-sialylated APOC3 proteoforms and sialylated proteoforms in overweight and obese people relative to healthy people.^176^ The Gordts group also demonstrated that a higher abundance of disialylated over monosialylated APOC3 correlates with lower triglyceride levels in plasma, highlighting the potential value of APOC3 glycoforms as biomarkers for evaluating diabetes and CVD risk.^177^ Those MS-based TDP studies shed new light on the functions of specific proteoforms of APOA1, APOA2, and APOC3 in modulating diabetes and CVD.

### 3.5 Infectious disease

MS-based TDP studies were previously implemented to explore potential mechanisms associated with pathogenic bacteria and virus-induced infectious disease and have been summarized in recent reviews.^178,179^ In recent years, SARS-CoV-2 has caused millions of deaths each year, calling for urgent action to study infection mechanisms, pathogenesis, and therapies. The glycosylated spike (S) protein on SARS-CoV-2 is essential in mediating viral infectivity. S protein RBD becomes the focus of many studies since it is the center region interacting with the angiotensin-converting enzyme 2 (ACE2) receptor on host cells and a promising target for therapeutics. The Ge group applied MS-based TDP to elucidate *O*-glycoform heterogeneity of SARS-CoV-2 and its Omicron variant on both structure and molecular signature aspects.^113,180^ In their studies, native TIMS-MS analysis enabled deciphering the structural heterogeneity of RBD proteoforms in SARS-CoV-2 and its Omicron variant samples; denaturing top-down MS on FTICR produced in-depth characterization of glycoforms and accurate localization of glycosylation sites.^113,180^ Several Omicron-specific *O*-glycoforms were identified for the first time, providing precious insight into the capability of escaping immunological protection.^180^ Additionally, different combinations of approaches have been explored to characterize highly complex glycoforms on RBD better. The Domínguez-Vega group carried out the structural and functional characterization of *O*- and *N*-glycosylation on RBD expressed in two different mammalian cells using CE-MS and matrix-assisted laser desorption ionization in source-decay (MALDI-ISD) MS.^181^ The Zhou group evaluated three different separation techniques (RPLC, CE, and hydrophilic interaction chromatography (HILIC)) for resolving glycoforms of intact recombinant RBDs and achieved 200 peaks using HILIC.^182^ Moreover, MS-based TDP is critical for evaluating immune responses after COVID-19 infection. Recent TDP mapping of immunoglobulin (Ig) repertoire by I2MS after COVID exposure or receiving vaccine documented that the I2MS workflow is robust for assessing immune responses to vaccination and pathogens.^183^

## 4. Conclusions and future directions

Biologists have widely accepted proteomics and applied it to basic and translational research.^184^ However, as the routine approach in proteomics, BUP has difficulties delineating proteoforms because an intact proteoform picture is lost during the enzymatic process. In the past decade, MS-intensive TDP has made tremendous progress in technological development for understanding protein functions in diseases and development *via* measuring intact proteoforms. Discovery and targeted TDP have been successfully applied to study the function of proteoforms in various diseases (*e.g.*, cancer, neurodegenerative diseases, cardiac injury, diabetes and cardiovascular diseases, and infectious diseases). TDP has discovered novel proteoform biomarkers of diseases.^185^ We expect that with further technological development of TDP, biologists will widely accept it to offer complementary information to BUP about the roles played by proteins in modulating biological processes.

Much more effort is needed to advance the technique of MS-intensive TDP to facilitate its broad adoption in biological research. Technical development is needed to boost proteome coverage, throughput, and quality of proteoform characterization. Integration of novel sample preparation methods, efficient liquid-phase separations, advanced MS (*e.g.*, charge detection MS, individual ion MS, and native MS), and better bioinformatics tools could be extremely valuable for solving the technical challenges of TDP. We must point out that high-quality and global top-down MS measurement of intact proteoforms, especially large and heavily modified ones, needs mass spectrometers with high speed, high mass resolution, and extensive proteoform gas-phase fragmentation. Those instrumentation are extremely expensive and have high maintenance costs, which is one critical factor limiting the broad use of MS-based TDP.

There are several new frontiers in the MS-intensive TDP field. First, increasing TDP applications are rolling from the conventional denaturing TDP into the native mode (native proteomics) to characterize protein complexes in cells.^48,186–191^ Second, single-cell TDP will be another hot research area to decipher cell-to-cell heterogeneity at the proteoform level.^27,88,89^ Third, the human proteoform project^185^ will be one focus of the TDP community. Fourth, deciphering proteoform function in biological processes and disease progression is essential for the TDP field. As mentioned in this review, many TDP studies have been done to better our understanding of protein function in various diseases. We expect more and more such studies to be carried out with better proteome coverage and proteoform characterization due to the continuous advancements of MS-based TDP techniques. Finally, combining MS-intensive TDP and BUP is crucial to advance TDP for high throughput and extensive characterization of proteoforms in complex samples. BUP data can provide rich information on types and sites of PTMs, which is extremely useful to interpret PTM combinations on proteoforms from TDP measurement. It is also essential to have a standardized procedure from sample preparation to database search, as the emerging field of TDP continues to develop.^68,147^ Unbiased quality control that singles out high-quality mass spectra is a gateway for building up TDP spectral libraries and, ultimately, the human proteoform atlas.^192^

## Data availability

All the necessary materials related to this work are included in the main manuscript.

## Conflicts of interest

The authors declare no competing financial interest.
